# *Mycobacterium tuberculosis *Rv0679c protein sequences involved in host-cell infection: Potential TB vaccine candidate antigen

**DOI:** 10.1186/1471-2180-10-109

**Published:** 2010-04-13

**Authors:** Diana P Cifuentes, Marisol Ocampo, Hernando Curtidor, Magnolia Vanegas, Martha Forero, Manuel E Patarroyo, Manuel A Patarroyo

**Affiliations:** 1 Fundación Instituto de Inmunología de Colombia, Carrera 50 No. 26-20, Bogotá, Colombia; 2 Universidad del Rosario, Calle 63 D No. 24-31, Bogotá, Colombia; 3 Universidad Nacional de Colombia, Carrera 45 No. 26-85, Bogotá, Colombia

## Abstract

**Background:**

To date, the function of many hypothetical membrane proteins of *Mycobacterium tuberculosis *is still unknown and their involvement in pathogen-host interactions has not been yet clearly defined. In this study, the biological activity of peptides derived from the hypothetical membrane protein Rv0679c of *M. tuberculosis *and their involvement in pathogen-host interactions was assessed. Transcription of the *Rv0679c *gene was studied in 26 *Mycobacterium *spp. Strains. Antibodies raised against putative B-cell epitopes of Rv0679c were used in Western blot and immunoelectron microscopy assays. Synthetic peptides spanning the entire length of the protein were tested for their ability to bind to A549 and U937 cells. High-activity binding peptides (HABPs) identified in Rv0679c were tested for their ability to inhibit mycobacterial invasion into cells.

**Results:**

The gene encoding Rv0679c was detected in all strains of the *M. tuberculosis *complex (MTC), but was only transcribed in *M. tuberculosis *H37Rv, *M. tuberculosis *H37Ra and *M. africanum*. Anti-Rv0679c antibodies specifically recognized the protein in *M. tuberculosis *H37Rv sonicate and showed its localization on mycobacterial surface. Four HABPs inhibited invasion of *M. tuberculosis *to target cells by up to 75%.

**Conclusions:**

The results indicate that Rv0679c HABPs and in particular HABP 30979 could be playing an important role during *M. tuberculosis *invasion of host cells, and therefore could be interesting research targets for studies aimed at developing strategies to control tuberculosis.

## Background

Tuberculosis (TB) is among the top three leading causes of death by a single infectious agent worldwide. The situation is further aggravated by the increased susceptibility of human immunodeficiency virus (HIV)-positive people to infection with *Mycobacterium tuberculosis *[1], and by the emergence of multidrug-resistant (MDR)-TB strains in many geographical areas [2]. An estimate of nearly 9.2 million cases of TB occurred during 2007, 4.1 million of which corresponded to new smear-positive cases and 14.8% were reported among HIV-positive people [3].

Unfortunately, the bacillus Calmette-Guérin (BCG) vaccine is insufficient to control the worldwide spread of this health threat, especially since it is contraindicated for HIV-positive people and has a variable efficacy, mostly due to its low capacity to stimulate the broad cell spectrum needed for inducing an effective immune response [4,5]. Therefore, a large body of research has focused on searching for new specific antigens of *M. tuberculosis *that could be used as new prophylactic alternatives with the aim of replacing or improving the currently available BCG vaccine [6-8].

The publication of the complete *M. tuberculosis *H37Rv genome sequence has opened a gate for the identification of genes that encode *M. tuberculosis *antigens putatively able to trigger an effective immune response and that could therefore be interesting as potential components of antituberculous subunit vaccines [9,10]. The immunological properties of these predicted *M. tuberculosis*-specific antigens have been characterized mainly using recombinant proteins [11]. Synthetic peptides have been also used with success for screening pathogen-specific genome regions of putative protective importance in order to identify T-cell reactivity [12]. In TB, synthetic peptides have shown good results for diagnosing TB in cattle [13] and in a protective vaccine tested in mice [14].

The first encounter between *M. tuberculosis *and the host cell occurs via an array of different receptor molecules, including complement receptors, the mannose receptor, the dendritic cell-specific intercellular adhesion molecule (ICAM)-3-grabbing nonintegrin (DC-SIGN), and Fc receptors [15]. The recent discovery of novel classes of receptors such as toll-like receptors, nucleotide-binding oligomerization domain (NOD)-like receptors, DC-SIGN, and Dectin-1, are giving clues about the possible host mechanisms involved in coordinating the innate and adaptive immune responses against *M. tuberculosis *[16]. Particularly, lipoproteins have been shown to trigger cytokine signaling via toll-like receptors on the surface of mammalian cells and therefore have been considered to be important effectors that may contribute to the pathogen's virulence. However, only a reduced number of predicted mycobacteriallipoproteins have been experimentally characterized [17].

Our institute has studied ligand-receptor interactions established between synthetic peptides derived from pathogen proteins and host-cell surface receptors, with the purpose of identifying high activity binding peptides (HABPs) involved in specific host-pathogen recognition interactions, and that could therefore be potential components of subunit vaccines. This methodology has been used and tested on different pathogens, including *Plasmodium falciparum, Plasmodium vivax *[18-20], Human papillomavirus [21] and Epstein-Barr virus [22], among others. Specifically in the case of *M. tuberculosis*, our group has characterized and determined the binding profiles of three mycobacterial membrane proteins [23-25]. More recently, the biological relevance of HABPs derived from some other mycobacterial proteins has been demonstrated using a flow-cytometry-based assay to assess the capacity of HABPs to mycobacterial inhibit invasion of target cells [26-28].

This study focused on the Rv0679c protein of *M. tuberculosis*, which is classified as a hypothetical membrane protein of the cell envelope. Its protein homolog in *M. bovis *BCG is a putative lipoprotein that has been shown to be tightly associated to lipoarabinomannan (LAM) [29], one of the major components of cell envelope involved in pro-inflammatory and anti-inflammatory responses [30]. The aim of the present study was to identify Rv0679c HABPs capable of inhibiting *M. tuberculosis *invasion of target cells that could therefore be considered as potential as candidate components for a chemically synthesized, subunit-based antituberculous vaccine.

## Methods

### Bioinformatics analysis

The sequence of the *M. tuberculosis *Rv0679c protein was downloaded from Tuberculist http://genolist.pasteur.fr/TubercuList/ and used as query sequence of a BLAST search http://www.ncbi.nlm.nih.gov/BLAST/. Type I and II signal peptides (typical of lipoproteins) were identified using LipoP 1.0 http://www.cbs.dtu.dk/services/LipoP/. Transmembrane regions were predicted using TMHMM v. 2.0 http://www.cbs.dtu.dk/services/TMHMM and TMPRED http://www.ch.embnet.org/software/TMPRED_form.html.

### Molecular assays

The presence and transcription of the *Rv0679c *gene was assessed in species and strains belonging to the *M. tuberculosis *complex and in mycobacteria other than tuberculosis. The following strains were tested (26 in total): *M. tuberculosis *H37Rv (ATCC 27294), *M. tuberculosis *H37Ra (ATCC 25177), *M. bovis *(ATCC 19210), *M. bovis *BCG (ATCC 35734), *M. africanum *(ATCC 25420), *M. microti *strain Pasteur (donated by Dr. Françoise Portaels), *M. flavescens *(ATCC 14474), *M. fortuitum *(ATCC 6841), *M. szulgai *(ATCC 35799), *M. peregrinum *(ATCC 14467), *M. phlei *(ATCC 11758), *M. scrofulaceum *(ATCC 19981), *M. avium *(ATCC 25291), *M. smegmatis *(ATCC 14468), *M. nonchromogenicum *(ATCC 19530), *M. simiae *(TMC 1595), *M. intracellulare *(ATCC 13950), *M. gastri *(ATCC 15754), *M. kansasii *(ATCC 12478), *M. dierhoferi *(ATCC 19340), *M. gordonae *(ATCC 14470), *M. marinum *(ATCC 927), *M. terrae *(ATCC 15755), *M. chelonae-chelonae *(ATCC 35752), *M. vaccae *(ATCC 15483), *M. triviale *(ATCC 23292). All mycobacterial strains were cultured for 5 to 15 days in Middlebrook 7H9 medium (Difco, New Jersey, USA) containing 0.05% Tween 80. Growth media were supplemented with oleic acid-albumin-dextrose-catalase (OADC) (Becton Dickinson, BBL; Sparks, MD) or ADC as needed. Genomic DNA isolated phenol-chloroform extraction, as described elsewhere [31]. PCR assays were carried out on a GeneAmp PCR System 9600 thermal cycler (Perkin-Elmer Life Sciences Inc., Boston, MA, USA) using 0.4 mM of direct (5'-CGCTACCCACTCCCG-3') and reverse primers (5'-CTTGTTGTTCGCACCAC-3') to amplify a 346-bp fragment of *Rv0679c*. Thermocycling conditions consisted of an initial denaturation at 94°C for 5 min, followed by 25 cycles according to the following conditions: 56°C for 30 s, 72°C for 40 s and 95°C for 40 s. A final 5 min extension step was performed at 72°C. Amplification products were separated in SYBR-stained 1% (*w*/*v*) agarose gels (Invitrogen).

For RT-PCR assays, RNA was isolated based on Katoch's methodology [32], assessing transcription of the *rpo*B housekeeping gene as positive transcription control [33].

### Detection of Rv0679c by Western blot and immunoelectron microscopy (IEM)

Expression of the *Rv0679c *gene was assessed by Western blot analysis of *M. tuberculosis *H37Rv sonicates using sera raised in goats obtained. Briefly, two goats (A-29 and B-86) nonreactive to *M. tuberculosis *H37Rv sonicate were inoculated with 5 mg of either polymerized forms of peptide 28528 (^43^CGTTTPATATTTTATSGPTAAPGC^62^) or peptide 28530 (^145^CGTYKNGDPTIDNLGAGNRINKEGC^165^), both in polymeric form and emulsified with Freund's incomplete adjuvant. These two peptides were chosen because the BepiPred 1.0b server http://www.cbs.dtu.dk/services/BepiPred/ predicted them as B cell epitopes. Subcellular localization was determined in a CM 10 transmission electron microscope (Philips, Suresne, Hauts-de-Seine, France), using thin slices (400 nm) of LR-White resin embedded mycobacteria. Goat anti-peptide sera were used as primary antibody and anti-goat IgG coupled to 10-nm colloidal gold particles as secondary antibody. Slices were stained with 6% uranyl acetate to enhance image contrast.

### Interaction of Rv0679c peptides with target cells

Nine nonoverlapping 20-mer-long peptides spanning the entire length of Rv0679c were synthesized and ^125^I-labeled according to previously described techniques [34-36]. Peptides were tested for their ability to bind to the A549 alveolar cell line (ATCC CLL-185) and to macrophages derived from U937 monocytes (ATCC CRL-2367). Briefly, 1.5 × 10^6 ^cells cultured in Roux flasks were dislodged using 1× Non-enzymatic Cell Dissociation Solution (Sigma) and incubated with increasing concentrations of ^125^I-labeled peptide (0-950 nM) in the presence or absence of unlabeled peptide (40 μM). Unbound peptide was removed using a dioctylphthalate-dibutylphthalate cushion, before measuring cell-associated radioactivity in a gamma counter (Gamma Counter Cobra II, Packard Instrument Co., Meriden, CT, USA).

Total binding minus nonspecific binding yielded the specific binding curve, whose slope corresponded to the binding activity of the peptide. Any peptide displaying a specific binding activity of ≥1% was considered a HABP [23-25,37]. Binding constants were determined by performing a saturation assay using U937 cells and peptide concentrations larger than the ones used for binding assays (0-4500 nM).

### Circular dichroism analyses of Rv0679c peptides

The secondary structure elements of the peptides spanning the entire length of Rv0679c were studied by circular dichroism. CD spectra of peptides (5 μM) dissolved in 30% trifluoroethanol (TFE) were acquired at 20°C by averaging three scans taken in a Jasco J-810 spectropolarimeter (wavelength range: 260-190 nm, scan rate: 20 nm/min, bandwidth: 1 nm), using a 1.00-cm pathway cuvette (Jasco Inc, Easton, MD). Data were corrected for baseline deviation [38]. The results were expressed as mean residue ellipticity [θ], the units being degrees × cm^2 ^× dmol^-1 ^according to the [Θ] = Θ_λ_/(100*lcn*) function, where θ_λ _is the measured ellipticity, *l *is the optical path length, *c *is the peptide concentration, and *n *is the number of residues in the amino acid sequence.

### Invasion inhibition assays

Rv0679c HABPs were assessed for their ability to inhibit mycobacterial invasion using a flow-cytometry-based assay developed by Bermúdez and Goodman [39] and later modified by us [26]. In brief, A549 and U937 cells (1 × 10^6^) seeded overnight on 6-well plates were incubated for 1 h with different peptide concentrations. SYBR-safe stained mycobacteria (10 × 10^6^) suspended in RPMI medium were added to each well (MOI: 1:10) and incubated overnight at 37°C. Inhibition controls consisted of Cytochalasin D (3 μM) or colchicine (50 μM). Extracellular bacilli were first inactivated by incubation with Amikacin (200 μg/mL) for 1 h and then removed by successive washes with Hanks Balanced Salt Solution (HBSS). Cells were dislodged from monolayers and stained with methylene blue for FACscan flow cytometry analysis (Becton Dickinson). The percentage of SYBR safe positive events in the flanked region were determined by registering a total of 30 000 events (only infected cells were detected on the FL1 channel due to their fluorescence characteristics). Data were statistically analyzed by applying a student's *t-*test.

### Internalization of latex beads

Internalization assays were carried out according to a methodology reported by El-Shazly and colleagues [40]. Briefly, A549 cells (1 × 10^6^) were exposed to peptide-coated fluorescent beads for 3 h. After removing noninternalized beads by washing cell thrice with HBSS, cells were dislodged from the monolayer and analyzed in a FACscan flow cytometer, same as described in invasion inhibition assays. The same assay was carried out using uncoated beads as negative control. An additional assay was carried out to determine whether the peptide alone enabled internalization of the latex beads by modifying the host cell membrane or whether internalization depended on the interaction between the peptide and the bead. For this assay, the control consisted on incubating cells for 2 h only with the peptide and then for 1 h with uncoated beads.

## Results

### Molecular analysis of the Rv0679c gene

**Figure 1:**
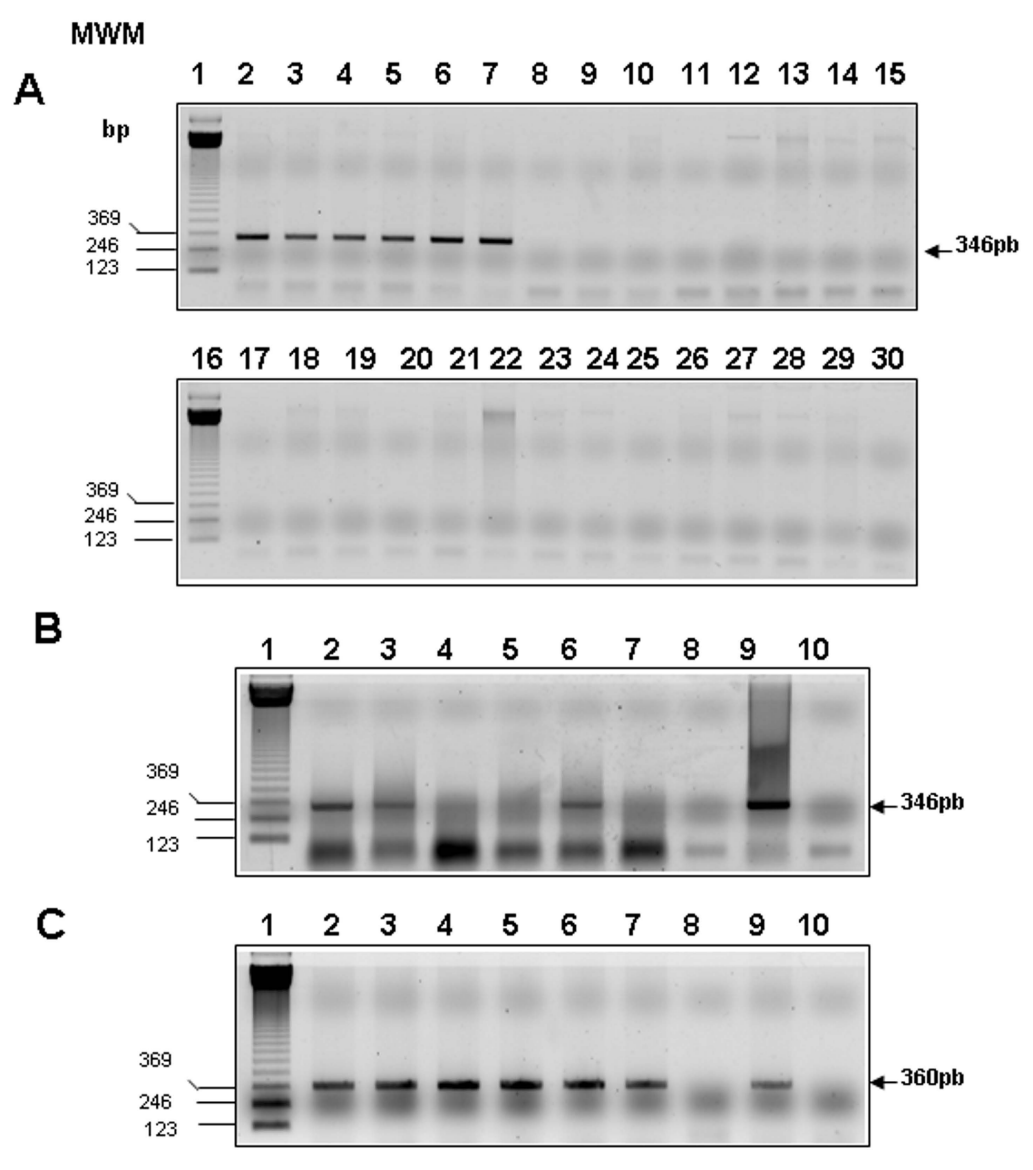
**Molecular assays**. **(A) **346-bp PCR product was only amplified from genomic DNA of species and strains belonging to the *M. tuberculosis *complex (MTC). (*Lane 1*) Molecular weight marker (MWM). (*Lane 2*) *M. tuberculosis *H37Rv. (*Lane 3*) *M. tuberculosis *H37Ra (ATCC 25177). (*Lane 4*) *M. bovis*. (*Lane 5*) *M. bovis *BCG. (*Lane 6*) *M. africanum*. (*Lane 7*) *M. microti *strain Pasteur. (*Lane 8*) *M. flavescens*. (*Lane 9*). *M. fortuitum*. (*Lane 10*) *M. szulgai*. (*Lane 11*) *M. peregrinum*. (*Lane 12*) *M. phlei*. (*Lane 13*) *M. scrofulaceum*. (*Lane 14*) *M. avium*. (*Lane 15*) *M. smegmatis*. (*Lane 16*) MWM. (*Lane 17*) *M. nonchromogenicum*. (*Lane 18*) *M. simiae*. (*Lane 19*) *M. intracellulare*. (*Lane 20*) *M. gastri*. (*Lane 21*)*M. kansasii*. (*Lane 22*) *M. dierhoferi*. (*Lane 23*) *M. gordonae*. (*Lane 24*), *M. marinum*. (*Lane 25*) *M. terrae*. (*Lane 26*) *M. chelonae-*. (*Lane 27*) *M. vaccae*. (*Lane 28*) *M. triviale*. (*Lane 29*) PCR negative control. **(B) **Detection of *Rv0679c *transcription in the MTC by RT-PCR using primers specific for the 346-bp fragment. (*Lanes 1-7*) same as in panel A. (*Lane 8*) *M. tuberculosis *DNA treated with DNAse Q (Negative control). (*Lane 9*) PCR positive control (*M. tuberculosis *H37Rv DNA). (*Lane 10*) PCR negative control. **(C) **RT-PCR detection of *rpoB *transcript as positive transcription control in the same strains.

Two primers flanking the region encoding amino acids 10-125 of Rv0679c were designed and synthesized in order to determine whether the gene was present in strains of the *M. tuberculosis *complex (MTC). An amplification band of a 346-bp band was detected in *M. tuberculosis *H37Rv, *M. tuberculosis *H37Ra, *M. bovis*, *M. bovis *BCG, *M. africanum *and *M. microti *(Figure 1A, lanes 2-7, respectively), but not in the remaining *Mycobacterium *strains analyzed in this study. Similarly, cDNA reverse transcription with the same primers confirmed transcription of the gene in *M. tuberculosis *H37Rv, *M. tuberculosis *H37Ra and *M. africanum*, as indicated by the amplification of a single 346-bp band (Figure 1B, lanes 2, 3 and 7, respectively). No amplification was detected in *M. bovis*, *M. bovis *BCG and *M. microti*, therefore suggesting that the gene is not transcribed in these species despite being present in these species. Amplification of the 360-bp fragment corresponding to the housekeeping gene *rpo*B was evidenced in all strains (Figure 1C).

### Goat anti-Rv0679c antibodies specifically recognized bands of about 18 and 20 kDa on *M. tuberculosis *sonicate and localized the protein on the surface

**Figure 2:**
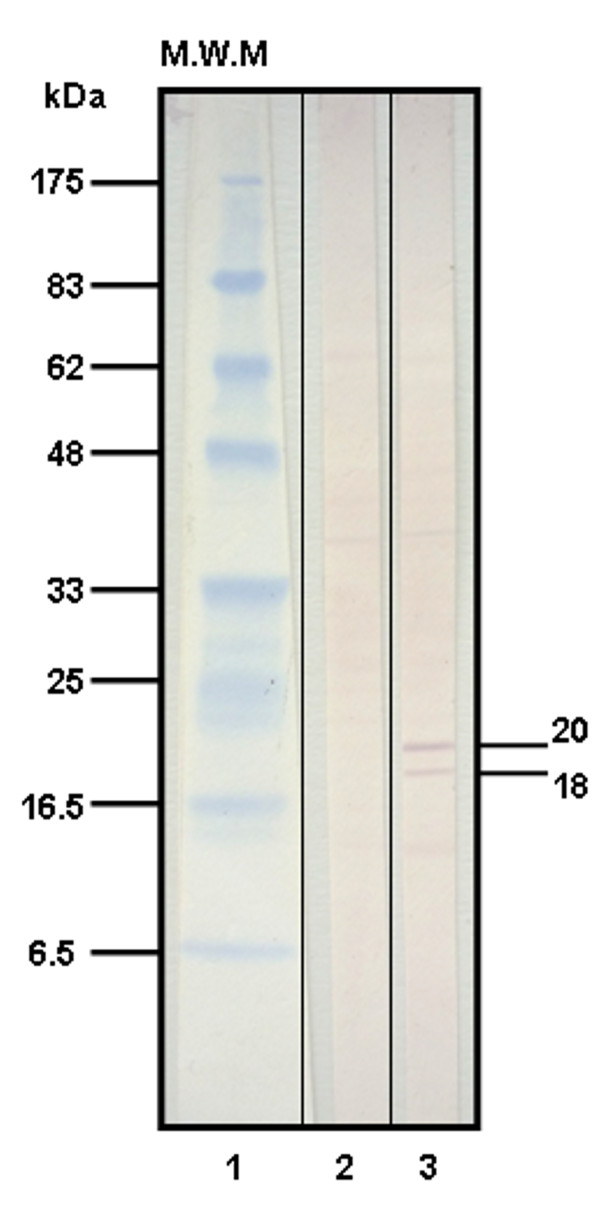
**Western blot analysis of *M. tuberculosis *H37Rv sonicate with goat B-86's serum raised against the polymerized Rv0679c peptide (CGTYKNGDPTIDNLGAGNRINKEGC)**. (*Lane 1*) Molecular weight marker (MWM). (*Lane 2*) Pre-immune serum. (*Lane 3*) Final bleeding serum. The image shows strong recognition of a 20-kDa band and a slighter recognition of an 18-kDa band by the final bleeding serum.

**Figure 3:**
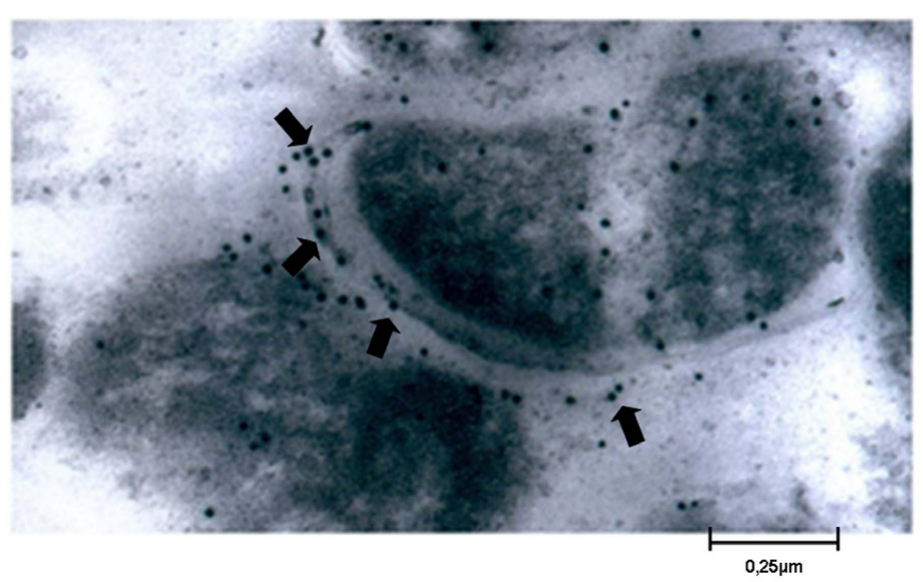
**Subcellular localization of the Rv0679c protein in *M. tuberculosis *H37Rv bacilli as assessed by IEM**. The arrows indicate the position of Rv0679c on mycobacterial surface. In this experiment, a 1:20 dilution of B-86 goat's serum was used as primary antibody and a 1:50 dilution of 10-nm gold-labeled anti-goat IgG as a secondary antibody.

Recognition of native Rv0679c protein in *M. tuberculosis *sonicate by antibodies raised in goat against the two polymerized synthetic peptides of Rv0679c was assessed by Western blot (Figure 2). Serum raised against polymerized peptide 28530 in the B-86 goat recognized two bands in *M. tuberculosis *sonicate with apparent molecular weights of 18 and 20 kDa (Figure 2, lane 3), of which the molecular mass of the first band is more in agreement with the molecular mass predicted for Rv0679c based on nucleotide sequence (16.6 kDa). According to IEM studies performed using the same serum, Rv0679c is most likely located on mycobacterial surface since the vast majority of gold particles were detected on the bacilli surface (see black arrows in Figure 3), whereas no immunolabeling was observed when the pre-immune serum was used (data not shown).

### Binding of Rv0679c peptides to U937 and A549 cells

**Figure 4:**
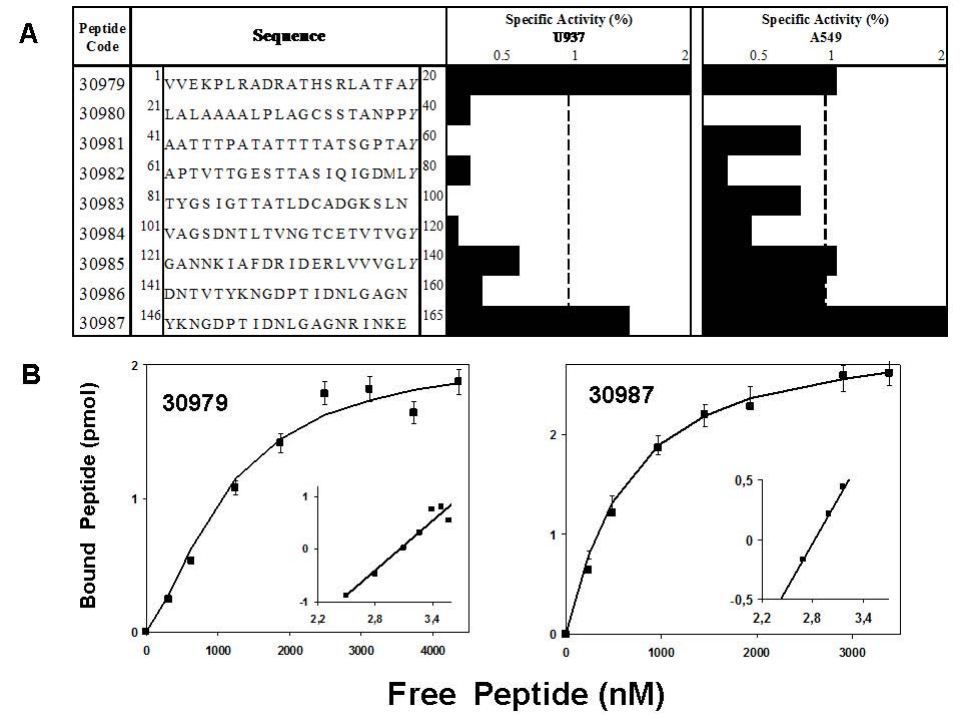
**Interaction of Rv0679c peptides with target cells**. **(A) **Binding profiles of peptides derived from Rv0679c to A549 and U937 cells. The slope of the specific binding curve is represented by the length of the black horizontal bars shown in front of each peptide sequence. Peptides showing a slope ≥1% were considered to be HABPs. Numbers shown in the first column correspond to our institute's serial numbering system. Superscripts at the beginning and end of the sequence indicate the peptide amino acid position within the protein. **(B) **Saturation binding curves for HABPs 30979 and 30987 binding with high activity to U937 cells. Saturation curves were obtained by plotting the specifically bound ^125^I-HABP concentration versus free ^125^I-HABP. Affinity constants and the maximum number of binding sites per cell were obtained from these curves. Inset: the abscissa is log F in the Hill plot and the ordinate is log [*B*/(*B*_m _- *B*)], where *B*_m _is the maximum amount of bound peptide, *B *is the amount of bound peptide and *F *is the amount of free peptide.

A highly specific binding assay was used to evaluate ligand-receptor interactions established between Rv0679c peptides and A549 and U937 cell surface receptors, same as has been reported for other mycobacterial proteins [23-25,37]. Based on this methodology, two HABPs binding with high activity to both cell lines were identified (namely HABPs 30979 and 30987), while other two HABPs (30985 and 30986) bound only to A549 cells. Figure 4a shows the sequences of Rv0679c synthetic peptides with their corresponding binding activities to A549 and U937 cells. All HABPs identified in Rv0679c were located toward the protein's C-terminus, except for HABP 30979 which was localized in the N-terminal end.

Rv0679c HABPs 30979 and 30987 were assessed by means of a saturation assay using concentrations of radiolabeled peptide larger than the ones used in conventional binding assays in order to determine dissociation constants (*K*_d_), Hill coefficients (*n*_H_) and approximate number of binding sites per cell (Figure 4b). The results showed that binding of these HABPs to surface receptors of U937 cells was saturable and of cooperative nature (*n*_H _= 1.50 for HABP 30979 and *n*_H _= 1.12 for HABP 30987). A dissociation constant of 1,100 nM and about 1.0 × 10^6 ^binding sites per cell were identified for HABP 30979, while HABP 30987 showed a dissociation constant of 600 nM and about 1.8 × 10^6 ^binding sites per cell.

### Secondary structure analyses of Rv0679c peptides by circular dichroism

**Figure 5:**
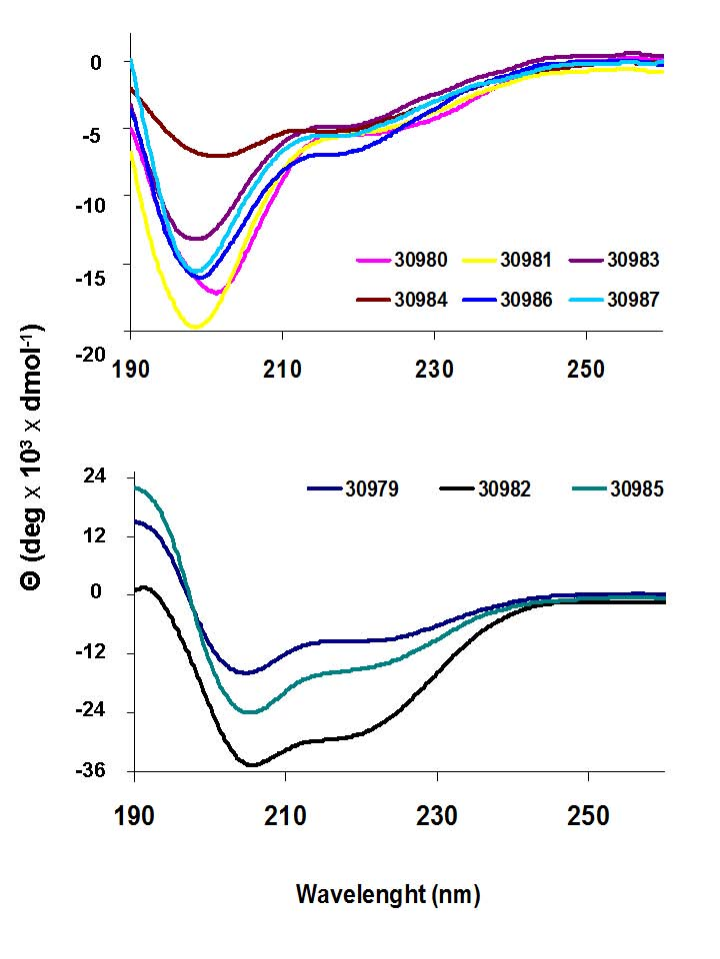
**CD spectra of Rv0679c peptides**. HABPS spectra were grouped in order to enable scale appreciation. Spectra were obtained by averaging three scans taken at 0.1 nm intervals from 260-190 nm at 20°C. [Θ] is the mean residue ellipticity per amino acid residue in the peptide. CD resolution: 0.1 millidegree (at ± 2.000 mdeg).

CD spectra of Rv0679c peptides obtained in 30% TFE are shown in Figure 5. The spectra of peptides 30982 and 30987 showed random coil structures, while the spectra of peptides 30979, 30981 and 30985 were consistent with α-helical structures. The remaining peptides of Rv0679c (30980, 30983, 30984 and 30986) displayed θ_λ _values not related to any defined structures.

### Inhibition of *M. tuberculosis *H37Rv invasion into A549 and U937 cells

The ability of Rv0679c HABPs to block mycobacterial entrance into A549 and U937 cells was evaluated using a flow-cytometry-based assay. Rv0679c peptides analyzed in such assay included peptides 30979 and 30987, which had been identified as HABPs for both cell lines, peptides 30985 and 30986 which had been identified as HABPs for A549 cells, and a low activity binding peptide (30982) which was used as negative control. Invasion of U937 cells was significantly inhibited by HABPs 30985 and 30986, but neither of these two HABPs showed a clear dose-dependent inhibitory behavior. Peptides 30985 and 30986 showed some signs of cytotoxicity when they were used at the largest peptide concentration (200 μM), as indicated by the lost of a portion of the cell monolayer and an abrupt decrease in percentages of invasion inhibition. No other peptide showed cytotoxic effects.

**Figure 6:**
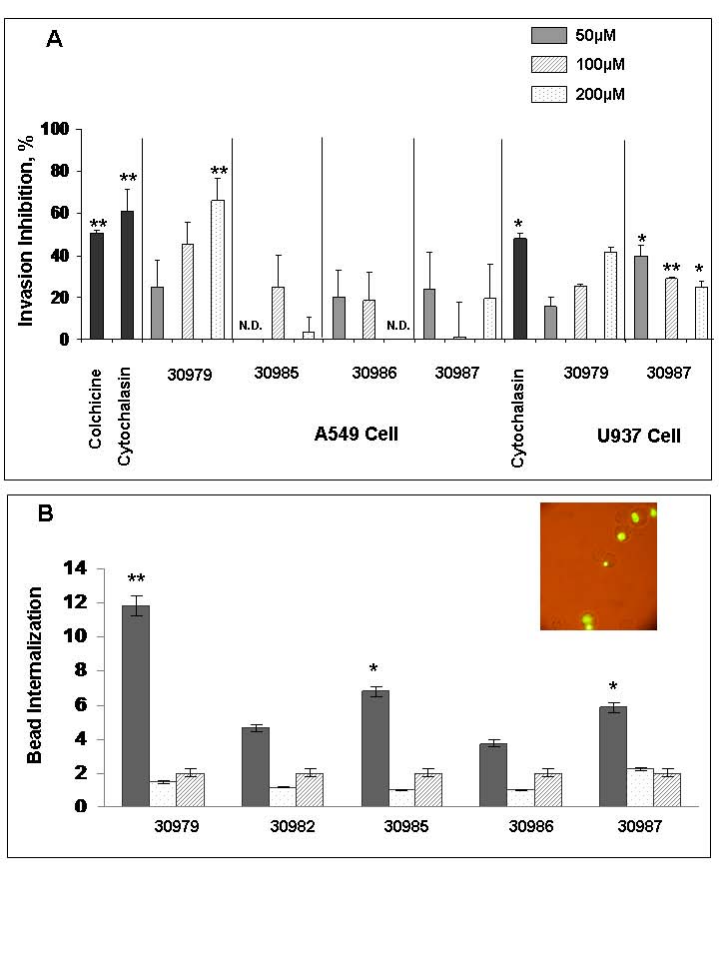
**Invasion inhibition and latex beads internalization assays**. **(A) **Results of invasion inhibition asssays performed with A549 and U937 cells and increasing concentrations of Rv0679c HABPs. **(B) **Internalization of peptide-coated beads by A549 epithelial cells. Dark gray columns correspond to the percentage of internalized peptide beads. Peptide 30982 was used as control. White columns correspond to the percentage of uncoated beads internalized when the assay was carried out incubating cells first with the peptide and then with uncoated latex beads. Striped columns correspond to the percentage of internalized beads when cells were incubated only with uncoated beads. *Inset: *latex beads internalized by A549 cells observed with fluorescence microscopy. The results correspond to the average invasion percentage calculated for each treatment ± standard deviations. *p ≤ 0.05; **p ≤ 0.01, according to a two-tailed student *t*-test.

HABPs 30985 to 30987 inhibited invasion of A549 cells by 20%, while HABP 30979 inhibited invasion of both cell lines in a dose-dependent manner. Moreover, the latter HABP inhibited invasion of U937 cells by a significantly larger percentage than the inhibition controls, whereas its inhibition ability in A549 cells was similar to the one shown by the controls. These results suggest that Rv0679c HABPs can prevent invasion of cells targeted by *M. tuberculosis *H37Rv. On the other hand, HABP 30987 inhibited invasion to U937 cells by a lower percentage compared to controls, but showed the highest inhibition percentage at the lowest peptide concentration used in this assay (Figure 6a). The negative control peptide did not inhibit cell invasion by mycobacteria (data not shown).

### Rv0679c HABPs 30986 and 30979 facilitate internalization of latex beads

A possible role for Rv0679c HABPs in host cell invasion was evaluated by determining their ability to facilitate internalization of fluorescent latex beads by A549 cells when beads are coated with these HABPs. Rv0679c peptides tested in this assay included 30979, 30985-30987, and peptide 30982 which was used as negative control. As it can be observed in Figure 6b, the highest internalization percentage was achieved when latex beads were coated with HABP 30979, followed by peptides 30985 and 30987. The percentage of internalization decreased when latex beads were coated with HABP 30986 compared to internalization of latex beads coated with the control peptide 30982. However, when cells were incubated first with each HABP and then with uncoated latex beads (control), smaller internalization percentages were found for all HABPs, and such percentages were smaller than the ones found when cells were incubated only with the beads (no peptide).

## Discussion

The mycobacterial cell envelope is a lipid-rich complex structure that surrounds the bacillus and is thought to play a critical role in the pathogenicity of *Mycobacterium tuberculosis*. Nearly 2.5% of the *M. tuberculosis *H37Rv proteome is predicted to consist of lipoproteins [17]. A large number of these mycobacterial lipoproteins have been suggested to be important components for the synthesis of the mycobacterial cell envelope, as well as for sensing processes, protection from stressful factors and host-pathogen interactions; nevertheless, the function and localization of a considerable number of putative lipoproteins remains yet unknown [41].

Lipoproteins are translocated across the cytoplasmic membrane and then anchored to either the periplasm or the outer membrane and have been suggested to play important roles related to virulence because they are predicted to participate in intracellular transport, cell-wall metabolism, cell adhesion, signaling and protein degradation [42]. Rv0679c was initially classified as a hypothetical membrane protein of *M. tuberculosis *[9] and was later suggested to be a putative lipoprotein [29]. It is a 165-amino-acid-long protein with a theoretical molecular mass of 16.6 kDa, whose function has not been fully characterized yet.

In this study, PCR and RT-PCR techniques were used to examine the distribution of the Rv0679c gene in the MTC, as well as in mycobacteria other than tuberculosis (which included saprophytic and environmental species), with the aim of establishing a preliminary relationship between the presence of the protein encoding gene in a particular mycobacterial species and its virulence, considering that to develop a subunit antituberculous vaccine, it would be better to select peptides (more specifically HABPs) from *M. tuberculosis *proteins involved in host cell invasion that are exclusively present in MTC or in mycobacterium species related to invasive processes or causing disease, such as Rv0679c. The results of this study indicate that the gene encoding Rv0679c is present in the MTC, as shown by the PCR amplification of a 346-bp band from genomic DNA of *M. tuberculosis *H37Rv, *M. tuberculosis *H37Ra, *M. africanum, M. bovis, M. bovis *BCG and *M. microti*; but no amplification was detected in *Mycobacterium *spp. strains outside the complex. Nevertheless, it is worth noting that *Rv0679c *homologues have been recently reported in different *Mycobacterium *genomes (e.g. *M. smegmatis, M. marinum *and *M. avium*), which indicates that such primers are specific for the MTC strains assessed in this study. Furthermore, reverse transcription assays indicate that the gene is actively transcribed in *M. tuberculosis *H37Rv, *M. tuberculosis *H37Ra and *M. africanum*. Intriguingly, although expression of Rv0679c homologous protein in *M. bovis *BCG was described by Matsuba *et al*. [29], gene transcription was not detected in *M. bovis *nor in *M. bovis *BCG in this study under normal culture conditions.

Once the presence and transcription of *Rv0679c *was determined in the MTC, the next step consisted in evaluating protein expression by Western blot analysis of *M. tuberculosis *H37Rv sonicate. Goat anti-Rv0679c peptide serum detected two bands of about 18 and 20 kDa, which differ from the theoretical molecular mass of 16.6 kDa predicted based on its amino acid composition. This slight difference could be caused by the post-translational modifications that lipoproteins undergo before reaching their destination as mature proteins, considering that pro-lipoproteins tend to be 2-3 kDa larger than mature lipoproteins [41].

According to bioinformatics predictions, Rv0679c lacks of transmembrane regions and contains an N-terminal signal sequence as well as a SPAse II cleavage site between residues 32-33, as indicated by the presence of a "lipobox" motif [LAGC] between amino acids 30-33. The presence of a signal peptide detected by using SignalP suggests that this protein is secreted via the Sec-dependent pathway, and is probably targeted by the lipobox motif to membrane surface where it remains attached by hydrophobic interactions. Briefly, after Rv0679c is translocated across the cytoplasmic membrane, the Cys residue of the lipobox motif is linked to a diacylglyceryl moiety. Then, a signal II peptidase cleaves off the signal peptide and the protein is anchored to the mycobacterial membrane via the diacylglyceryl moiety [41]. These computational predictions are in agreement with the cellular localization observed in IEM studies in which the protein was detected on the surface of *M. tuberculosi*s H37Rv bacilli.

To determine whether the peptides comprising Rv0679c established ligand-receptor interactions with *M. tuberculosi*s susceptible human host cells, binding assays were performed with the U937 phagocytic and A549 epithelial cell lines. HABPs 30985 to 30987 comprising amino acids 121-165 showed higher binding activities to receptors on the surface of epithelial cells, whereas their binding activities to the phagocytic line were lower. Such differential binding behavior may be caused by differences between the surface receptors expressed by each cell line or their distinct physiological functions.

Interestingly, Rv0679c HABPs 30985, 30986 and 30987 are consecutively positioned within the protein's C-terminus, suggesting that the region formed by these three HABPs is implicated in binding of *M. tuberculosis *to target cells. Also, the Hill analysis showed high binding affinity interactions with a large number of receptor molecules on the surface of U937 cells, as indicated by their dissociation constant within the nanomolar range. Moreover, the formation of ligand-receptor complexes appears to facilitate binding of more HABPs, as shown by the positive Hill coefficient.

All HABPs tested in invasion inhibition assays prevented cell invasion by *M. tuberculosis *by a larger or comparable percentage, compared to the colchicine and Cytochalasin D controls. Regarding HABP 30986, an inhibitory effect similar to the one shown by HABPs 30985 and 30987 was observed on A549 cells at all concentrations used in this assay. Moreover, HABP 30987 showed larger inhibitory effect at the smaller concentration tested in this assay. HABP 30979 inhibited invasion of both cell types by a larger or even higher percentage than the ones shown by the colchicine and Cytochalasin controls. This HABP showed a dose-dependent inhibitory effect on both cells, achieving the highest inhibitory percentage at 200 μM.

The ability of Rv0679c peptides to inhibit *M. tuberculosis *invasion of target cells suggests that active and specific binding to cell surface receptors prevents entry of *M. tuberculosis *through this invasion pathway. Such notion is further supported by the results of internalization assays carried out with peptide-coated latex beads and epithelial cells, where peptide-coated beads were more actively internalized than uncoated beads. Particularly HABP 30979, which was the strongest invasion inhibitor, displayed the highest internalization percentages.

On the other hand, the large inhibition percentages obtained with phagocytic cells in comparison to the ones obtained with epithelial cells might be explained by the cooperativity phenomenon observed in saturation assays with the phagocytic cell line, since the amount of peptide that binds to surface receptors is proportional to the probability of forming more stable ligand-receptor complexes and thereby of restricting mycobacterial entrance. Furthermore, since some HABPs showed high binding activity to one cell type but low binding activity to the other one, it could be suggested that peptide binding activity depends on specific receptor molecules expressed on each cell type. Consequently, binding of Rv0679c HABPs with high activity to both cell lines could be due to the presence of the same receptor on both cell types or to different receptors with similar characteristics.

To date, no structural model has been reported for this protein. Therefore, CD assays were conducted in order to determine whether there was a relationship between the secondary structure of Rv0679c peptides and their binding ability or in their ability to inhibit mycobacterial invasion. CD spectrum data suggested that the secondary structure of HABP 30979 and 30985 was formed by α-helix and random coil elements, while peptides 30982 to 30984 and HABPs 30986 and 30987 showed undefined structural features. The results indicate that there is not a direct relationship between the structure of HABPs and their ability to binding to target cells.

Interestingly, the results obtained in this study showed that the HABPs that inhibited mycobacterial invasion to target cells more efficiently were also the ones that showed the larger internalization percentages, therefore suggesting that Rv0679c HABPs promote entry of pathogenic *M. tuberculosis *into host cells. Specifically, the binding region formed by HABPs 30985-30987 at the protein's C-terminal region appears to have a key role during *M. tuberculosis *invasion.

The confirmation of Rv0679c's location in mycobacterial surface, together with the identification of a binding region formed by HABPs 30985-30987, suggest that this protein may be related to adhesion and/or invasion processes. In addition, such surface localization could be facilitating contact between the bacilli and its host cell, thereby leading to triggering the host's immune response via interaction with host cell surface receptors [16].

## Conclusions

The complexity of *Mycobacterium tuberculosis *as a pathogen and the variety of mechanisms that it uses for invading host cells makes it necessary to develop an effective strategy to block the invasion of target cells. Our proposal is based on searching for fragments of different proteins involved in the mycobacteria-host cell interaction. In our experience, sequences that bind specifically to target cells and that are capable of blocking invasion could be used as template to design peptides with ability to immunomodulate the protective response against tuberculosis. The immune response triggered against mycobacterial high-specific binding sequences could prevent invasion of target cells, either during a first encounter with the bacillum or during the reactivation of a latent infection.

It has been reported that a considerable number of secreted proteins are protective antigens and therefore have been considered as attractive candidates to develop subunit vaccines [43-46]. Moreover, they are hypothesized to mediate mycobacterial entry into the host cell [47].

Traditionally, vaccine development has been founded on the humoral immune response, which involves antibody production and is mainly targeted against extracellular microorganisms, whereas the immune response against intracellular microorganisms is mainly driven by cellular immune mechanisms. In addition, the distinction between the Th1 and Th2 cellular immune responses is complex for some of the antigens or immunogens included in vaccines that induce cellular as well as humoral immune responses, and it is not yet clear the degree of independence between antibody-mediated and cell-mediated immune responses under physiological conditions [48,49].

Considering the variety of broad interactions of B lymphocytes with cellular immunity, B cells could have a significant impact on the outcome of airborne challenge with *M. tuberculosis *as well as the resultant inflammatory response [49]. Therefore, we expect for peptides of Rv0679c to induce an immune response where humoral and cellular immunity are not mutually excluded.

The identification of Rv0679c HABPs capable of inhibiting target cell invasion by *M. tuberculosis *via host-cell receptor interactions supports their inclusion in further immunological studies in animal models aimed at evaluating their potential as components of a subunit-based antituberculous vaccine.

## Authors' contributions

DPC carried out molecular assays and drafted the manuscript. MO participated in the experimental design, data analysis and interpretation, and critically revised the manuscript. MAP participated in the experimental design and coordinated the study. HC carried out ligand-receptor assays. MV participated in the peptide synthesis. MF carried out immunoassays. MEP conceived and supervised the study. All authors read and approved the final manuscript.

## References

[B1] Aliyu MH Salihu HM Tuberculosis and HIV disease: two decades of a dual epidemic Wiener klinische Wochenschrift 2003 115 19-20 685 697 10.1007/BF03040884 14650943

[B2] Iseman MD Treatment and implications of multidrug-resistant tuberculosis for the 21st century Chemotherapy 1999 45 Suppl 2 34 40 10.1159/000048480 10449896

[B3] Global Tuberculosis Control, Epidemiology, Strategy, Financing http://www.who.int/tb/publications/global_report/2009/pdf/full_report.pdf

[B4] Batoni G Esin S Pardini M Bottai D Senesi S Wigzell H Campa M Identification of distinct lymphocyte subsets responding to subcellular fractions of Mycobacterium bovis bacille calmette-Guerin (BCG) Clinical and experimental immunology 2000 119 2 270 279 10.1046/j.1365-2249.2000.01137.x 10632662 PMC1905498

[B5] Hesseling AC Schaaf HS Hanekom WA Beyers N Cotton MF Gie RP Marais BJ van Helden P Warren RM Danish bacille Calmette-Guerin vaccine-induced disease in human immunodeficiency virus-infected children Clin Infect Dis 2003 37 9 1226 1233 10.1086/378298 14557968

[B6] Kaufmann SH Baumann S Nasser Eddine A Exploiting immunology and molecular genetics for rational vaccine design against tuberculosis Int J Tuberc Lung Dis 2006 10 10 1068 1079 17044198

[B7] Changhong S Hai Z Limei W Jiaze A Li X Tingfen Z Zhikai X Yong Z Therapeutic efficacy of a tuberculosis DNA vaccine encoding heat shock protein 65 of *Mycobacterium tuberculosis *and the human interleukin 2 fusion gene Tuberculosis (Edinburgh, Scotland) 2009 89 1 54 61 10.1016/j.tube.2008.09.005 19056317

[B8] Romano M Rindi L Korf H Bonanni D Adnet PY Jurion F Garzelli C Huygen K Immunogenicity and protective efficacy of tuberculosis subunit vaccines expressing PPE44 (Rv2770c) Vaccine 2008 26 48 6053 6063 10.1016/j.vaccine.2008.09.025 18822333

[B9] Cole ST Brosch R Parkhill J Garnier T Churcher C Harris D Gordon SV Eiglmeier K Gas S Barry CE Deciphering the biology of *Mycobacterium tuberculosis *from the complete genome sequence Nature 1998 393 6685 537 544 10.1038/31159 9634230

[B10] Chakravarti DN Fiske MJ Fletcher LD Zagursky RJ Application of genomics and proteomics for identification of bacterial gene products as potential vaccine candidates Vaccine 2000 19 6 601 612 10.1016/S0264-410X(00)00256-5 11090710

[B11] Mustafa A LT S Progress towards the development of new anti-tuberculosis vaccines Focus on Tuberculosis Research 2005 New York, USA 47 76

[B12] Arend SM Geluk A van Meijgaarden KE van Dissel JT Theisen M Andersen P Ottenhoff TH Antigenic equivalence of human T-cell responses to *Mycobacterium tuberculosis *-specific RD1-encoded protein antigens ESAT-6 and culture filtrate protein 10 and to mixtures of synthetic peptides Infection and immunity 2000 68 6 3314 3321 10.1128/IAI.68.6.3314-3321.2000 10816479 PMC97589

[B13] Vordermeier HM Whelan A Cockle PJ Farrant L Palmer N Hewinson RG Use of synthetic peptides derived from the antigens ESAT-6 and CFP-10 for differential diagnosis of bovine tuberculosis in cattle Clinical and diagnostic laboratory immunology 2001 8 3 571 578 11329460 10.1128/CDLI.8.3.571-578.2001 PMC96103

[B14] Olsen AW Hansen PR Holm A Andersen P Efficient protection against *Mycobacterium tuberculosis *by vaccination with a single subdominant epitope from the ESAT-6 antigen European journal of immunology 2000 30 6 1724 1732 10.1002/1521-4141(200006)30:6<1724::AID-IMMU1724>3.0.CO;2-A 10898510

[B15] Ernst JD Macrophage receptors for *Mycobacterium tuberculosis* Infection and immunity 1998 66 4 1277 1281 9529042 10.1128/iai.66.4.1277-1281.1998 PMC108049

[B16] Jo EK Mycobacterial interaction with innate receptors: TLRs, C-type lectins, and NLRs Current opinion in infectious diseases 2008 21 3 279 286 10.1097/QCO.0b013e3282f88b5d 18448973

[B17] Sutcliffe IC Harrington DJ Lipoproteins of *Mycobacterium tuberculosis *: an abundant and functionally diverse class of cell envelope components FEMS microbiology reviews 2004 28 5 645 659 10.1016/j.femsre.2004.06.002 15539077

[B18] Curtidor H Rodriguez LE Ocampo M Lopez R Garcia JE Valbuena J Vera R Puentes A Vanegas M Patarroyo ME Specific erythrocyte binding capacity and biological activity of *Plasmodium falciparum *erythrocyte binding ligand 1 (EBL-1)-derived peptides Protein Sci 2005 14 2 464 473 10.1110/ps.041084305 15659376 PMC2254251

[B19] Ocampo M Rodriguez LE Curtidor H Puentes A Vera R Valbuena JJ Lopez R Garcia JE Ramirez LE Torres E Identifying *Plasmodium falciparum *cytoadherence-linked asexual protein 3 (CLAG 3) sequences that specifically bind to C32 cells and erythrocytes Protein Sci 2005 14 2 504 513 10.1110/ps.04883905 15659379 PMC2253410

[B20] Rodriguez LE Urquiza M Ocampo M Curtidor H Suarez J Garcia J Vera R Puentes A Lopez R Pinto M *Plasmodium vivax *MSP-1 peptides have high specific binding activity to human reticulocytes Vaccine 2002 20 9-10 1331 1339 10.1016/S0264-410X(01)00472-8 11818151

[B21] Vera-Bravo R Ocampo M Urquiza M Garcia JE Rodriguez LE Puentes A Lopez R Curtidor H Suarez JE Torres E Human papillomavirus type 16 and 18 L1 protein peptide binding to VERO and HeLa cells inhibits their VLPs binding International journal of cancer 2003 107 3 416 424 10.1002/ijc.11433 14506742

[B22] Urquiza M Suarez J Lopez R Vega E Patino H Garcia J Patarroyo MA Guzman F Patarroyo ME Identifying gp85-regions involved in Epstein-Barr virus binding to B-lymphocytes Biochemical and biophysical research communications 2004 319 1 221 229 10.1016/j.bbrc.2004.04.177 15158465

[B23] Vera-Bravo R Torres E Valbuena JJ Ocampo M Rodriguez LE Puentes A Garcia JE Curtidor H Cortes J Vanegas M Characterising *Mycobacterium tuberculosis *Rv1510c protein and determining its sequences that specifically bind to two target cell lines Biochemical and biophysical research communications 2005 332 3 771 781 10.1016/j.bbrc.2005.05.018 15907793

[B24] Forero M Puentes A Cortes J Castillo F Vera R Rodriguez LE Valbuena J Ocampo M Curtidor H Rosas J Identifying putative *Mycobacterium tuberculosis *Rv2004c protein sequences that bind specifically to U937 macrophages and A549 epithelial cells Protein Sci 2005 14 11 2767 2780 10.1110/ps.051592505 16199660 PMC2253216

[B25] Garcia J Puentes A Rodriguez L Ocampo M Curtidor H Vera R Lopez R Valbuena J Cortes J Vanegas M *Mycobacterium tuberculosis *Rv2536 protein implicated in specific binding to human cell lines Protein Sci 2005 14 9 2236 2245 10.1110/ps.051526305 16131654 PMC2253470

[B26] Chapeton-Montes JA Plaza DF Curtidor H Forero M Vanegas M Patarroyo ME Patarroyo MA Characterizing the *Mycobacterium tuberculosis *Rv2707 protein and determining its sequences which specifically bind to two human cell lines Protein Sci 2008 17 2 342 351 10.1110/ps.073083308 18096644 PMC2222728

[B27] Chapeton-Montes JA Plaza DF Barrero CA Patarroyo MA Quantitative flow cytometric monitoring of invasion of epithelial cells by *Mycobacterium tuberculosis* Front Biosci 2008 13 650 656 10.2741/2709 17981577

[B28] Patarroyo MA Plaza DF Ocampo M Curtidor H Forero M Rodriguez LE Patarroyo ME Functional characterization of *Mycobacterium tuberculosis *Rv2969c membrane protein Biochemical and biophysical research communications 2008 372 4 935 940 10.1016/j.bbrc.2008.05.157 18539140

[B29] Matsuba T Suzuki Y Tanaka Y Association of the Rv0679c protein with lipids and carbohydrates in *Mycobacterium tuberculosis*/*Mycobacterium bovis *BCG Archives of microbiology 2007 187 4 297 311 10.1007/s00203-006-0195-4 17252234

[B30] Briken V Porcelli SA Besra GS Kremer L Mycobacterial lipoarabinomannan and related lipoglycans: from biogenesis to modulation of the immune response Molecular microbiology 2004 53 2 391 403 10.1111/j.1365-2958.2004.04183.x 15228522

[B31] Del Portillo P Murillo LA Patarroyo ME Amplification of a species-specific DNA fragment of *Mycobacterium tuberculosis *and its possible use in diagnosis Journal of clinical microbiology 1991 29 10 2163 2168 1939567 10.1128/jcm.29.10.2163-2168.1991 PMC270291

[B32] Katoch VM Cox RA Step-wise isolation of RNA and DNA from mycobacteria Int J Lepr Other Mycobact Dis 1986 54 3 409 415 2427627

[B33] Lee H Park HJ Cho SN Bai GH Kim SJ Species identification of mycobacteria by PCR-restriction fragment length polymorphism of the rpoB gene Journal of clinical microbiology 2000 38 8 2966 2971 10921960 10.1128/jcm.38.8.2966-2971.2000 PMC87161

[B34] Houghten RA General method for the rapid solid-phase synthesis of large numbers of peptides: specificity of antigen-antibody interaction at the level of individual amino acids Proceedings of the National Academy of Sciences of the United States of America 1985 82 15 5131 5135 10.1073/pnas.82.15.5131 2410914 PMC390513

[B35] Tam JP Heath WF Merrifield RB SN 1 and SN 2 mechanisms for the deprotection of synthetic peptides by hydrogen fluoride. Studies to minimize the tyrosine alkylation side reaction International journal of peptide and protein research 1983 21 1 57 65 6826283 10.1111/j.1399-3011.1983.tb03078.x

[B36] Yamamura H Enna S Kuhar M Neurotransmitter receptor binding 1978 New York: Raven Press

[B37] Plaza DF Curtidor H Patarroyo MA Chapeton-Montes JA Reyes C Barreto J Patarroyo ME The *Mycobacterium tuberculosis *membrane protein Rv2560--biochemical and functional studies The FEBS journal 2007 274 24 6352 6364 18005255 10.1111/j.1742-4658.2007.06153.x

[B38] Sreerama N Venyaminov SY Woody RW Estimation of the number of alpha-helical and beta-strand segments in proteins using circular dichroism spectroscopy Protein Sci 1999 8 2 370 380 10048330 10.1110/ps.8.2.370 PMC2144265

[B39] Bermudez LE Goodman J *Mycobacterium tuberculosis *invades and replicates within type II alveolar cells Infection and immunity 1996 64 4 1400 1406 8606107 10.1128/iai.64.4.1400-1406.1996 PMC173932

[B40] El-Shazly S Ahmad S Mustafa AS Al-Attiyah R Krajci D Internalization by HeLa cells of latex beads coated with mammalian cell entry (Mce) proteins encoded by the mce3 operon of *Mycobacterium tuberculosis* Journal of medical microbiology 2007 56 Pt 9 1145 1151 10.1099/jmm.0.47095-0 17761475

[B41] Rezwan M Grau T Tschumi A Sander P Lipoprotein synthesis in mycobacteria Microbiology (Reading, England) 2007 153 Pt 3 652 658 17322184 10.1099/mic.0.2006/000216-0

[B42] Nguyen KT Piastro K Derbyshire KM LpqM, a mycobacterial lipoprotein-metalloproteinase, is required for conjugal DNA transfer in *Mycobacterium smegmatis* Journal of bacteriology 2009 191 8 2721 2727 10.1128/JB.00024-09 19233923 PMC2668431

[B43] Andersen P Askgaard D Ljungqvist L Bennedsen J Heron I Proteins released from *Mycobacterium tuberculosis *during growth Infect Immun 1991 59 6 1905 1910 1903768 10.1128/iai.59.6.1905-1910.1991 PMC257941

[B44] Andersen P Askgaard D Ljungqvist L Bentzon MW Heron I T-cell proliferative response to antigens secreted by *Mycobacterium tuberculosis* Infect Immun 1991 59 4 1558 1563 1900811 10.1128/iai.59.4.1558-1563.1991 PMC257876

[B45] Horwitz MA Lee BW Dillon BJ Harth G Protective immunity against tuberculosis induced by vaccination with major extracellular proteins of *Mycobacterium tuberculosis* Proceedings of the National Academy of Sciences of the United States of America 1995 92 5 1530 1534 10.1073/pnas.92.5.1530 7878014 PMC42553

[B46] Orme IM Induction of nonspecific acquired resistance and delayed-type hypersensitivity, but not specific acquired resistance in mice inoculated with killed mycobacterial vaccines Infect Immun 1988 56 12 3310 3312 3141288 10.1128/iai.56.12.3310-3312.1988 PMC259741

[B47] Garcia-Perez BE Mondragon-Flores R Luna-Herrera J Internalization of *Mycobacterium tuberculosis *by macropinocytosis in non-phagocytic cells Microb Pathog 2003 35 2 49 55 10.1016/S0882-4010(03)00089-5 12901843

[B48] Igietseme JU Eko FO He Q Black CM Antibody regulation of Tcell immunity: implications for vaccine strategies against intracellular pathogens Expert review of vaccines 2004 3 1 23 34 10.1586/14760584.3.1.23 14761241

[B49] Maglione PJ Chan J How B cells shape the immune response against *Mycobacterium tuberculosis* Eur J Immunol 2009 39 3 676 686 10.1002/eji.200839148 19283721 PMC2760469

